# Role of temperature in reported chickenpox cases in northern European countries: Denmark and Finland

**DOI:** 10.1186/s13104-018-3497-0

**Published:** 2018-06-13

**Authors:** Ayako Sumi

**Affiliations:** Department of Hygiene, Sapporo Medical University School of Medicine, S-1, W-17, Chuo-ku, Sapporo, Hokkaido 060-8556 Japan

**Keywords:** Chickenpox, Seasonality, Surveillance, Temperature, Time-series analysis

## Abstract

**Objective:**

In this study, we sought to explore the temperature-dependent transition of patterns of reported chickenpox cases in the northern European countries of Denmark and Finland to help determine the potential relationship with epidemiological factors of the disease. We performed time-series analysis consisting of a spectral analysis based on the maximum entropy method in the frequency domain and the nonlinear least squares method in the time domain, using the following time-series data: monthly data of reported chickenpox cases and mean temperatures in the pre-vaccination era for Denmark and Finland. The results were compared with those reported for China and Japan in our previous studies.

**Results:**

Time-series data of chickenpox cases for both Denmark and Finland showed a peak each winter, resulting in a unimodal cycle. For investigating the origin of the unimodal cycle, we set the contribution ratio of the 1-year cycle, *Q*_1_, as the contribution of the amplitude of a 1-year cycle, to the entire amplitude of the time-series data. The *Q*_1_ values for both countries clearly showed a positive correlation with the annual mean temperature of each country. The mean temperature substantially influenced the incidence of chickenpox in both countries.

**Electronic supplementary material:**

The online version of this article (10.1186/s13104-018-3497-0) contains supplementary material, which is available to authorized users.

## Introduction

Chickenpox is a common infectious disease that remains an important public health problem worldwide [1–4]. Potential transmission of chickenpox is thought to be affected by climate change, and some studies have investigated the relationship between weather variability and the occurrence of infectious diseases [4–6]. We previously demonstrated that temperature was related to reported chickenpox incidence in Japan [3]. Therein, bimodal cycles of reported chickenpox incidences that were clearly observed in northern Japan disappeared at lower latitudes, and unimodal cycles appeared in southern Japan. This transition of patterns of reported chickenpox incidences in Japan was thought to depend on temperature.

More recently, we indicated that, for subtropical Wuhan and Hong Kong in China, both located south of the southernmost point of Japan, mean temperature and rainfall have a considerable influence on the incidence of chickenpox; we suggested that the epidemiology of chickenpox in Wuhan and Hong Kong differs from that in temperate Japan [7]. The effect of meteorological factors on chickenpox incidence may differ from one country to another in different climatic regions. To investigate the reasons underlying causes of chickenpox virus transmission in each climatic region, a systematic study is necessary; the investigation should quantify the impact of meteorological factors on chickenpox incidence in different countries in each climatic region.

In this study, we investigated whether the temperature-dependent transition of patterns of reported chickenpox incidences in Japan [3] also applies to northern European areas, such as Denmark and Finland, which are located north of the northernmost point of Japan and have good historical records of chickenpox as a notifiable disease [8]. The present method of analysis consisted of spectral analyses based on the maximum entropy method (MEM) in the frequency domain and the least squares method (LSM) in the time domain [3, 6, 9–11]. The present results provided additional results to our previous works [3, 7]. The results presented herein were compared with the cases of Japan [3], and Wuhan and Hong Kong [7].

## Main text

### Temporal variations in chickenpox data and temperature data

Monthly time series data of chickenpox case notifications and mean temperature in Denmark and Finland in the pre-vaccination era were used in the present study: the data from January 1938 to December 1960 were used for Denmark [12] and data from January 1952 to December 1970 were used for Finland [13, 14].

**Fig. 1:**
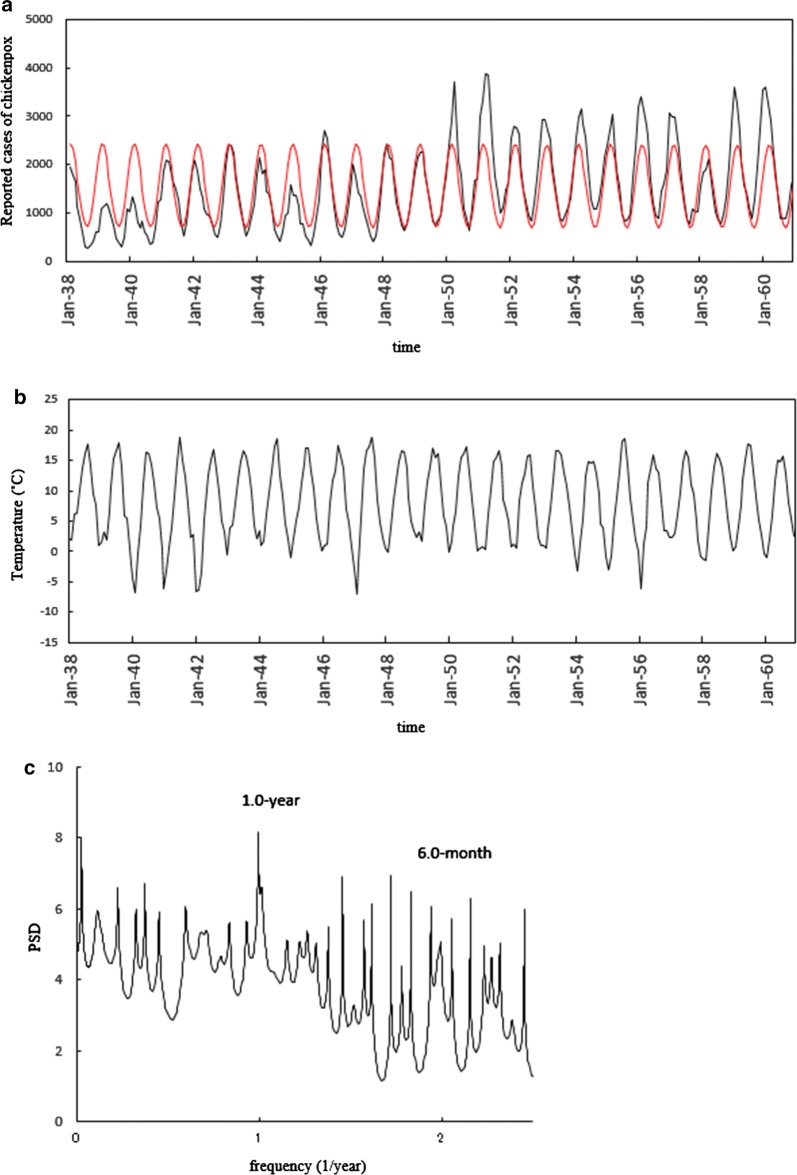
Seasonality of the monthly reported number for chickenpox cases in Denmark from January 1938 to December 1960. **a** Comparison of the least-squares fitting curves calculated for 1.0- and 0.5-year periodic modes (red line) with chickenpox data (solid line). **b** Monthly data of the mean temperature (°C). **c** Power spectral density of the chickenpox data

**Fig. 2:**
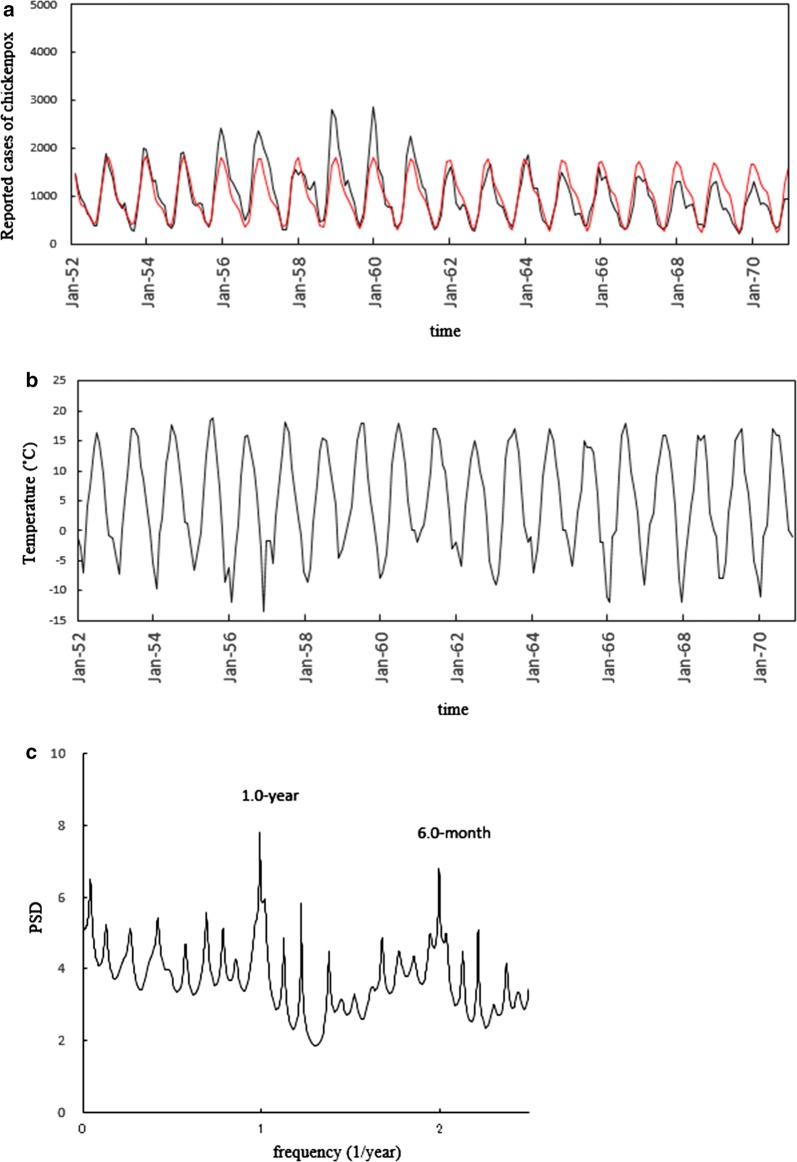
Seasonality of the monthly reported number for chickenpox cases in Finland from January 1952 to December 1970. **a** Comparison of the least-squares fitting curves calculated for 1.0- and 0.5-year periodic modes (red line) with chickenpox data (solid line). **b** Monthly data of the mean temperature (°C). **c** Power spectral density of the chickenpox data

Figure 1 indicates the monthly reported chickenpox data and the temperature data for Denmark (a and b, respectively), and Fig. 2 indicates the equivalent data for Finland (a and b, respectively). For the chickenpox and temperature data of both countries (Figs. 1 and 2), annual cycles were clearly observed, and these were modulated by irregular, shorter-term variations within the 1-year cycle.

For Denmark, peaks in the chickenpox data (Fig. 1a) were observed in January, February and March. The temporal pattern in temperature (Fig. 1b) revealed an increase, peaking at a maximum in summer (June–August), followed by a decrease, reaching a minimum in winter (December–March). For Finland, peaks in the chickenpox data (Fig. 2a) were observed in November, December and January. The temporal pattern in temperature (Fig. 2b) revealed an increase, peaking at a maximum in summer (June–August), followed by a decrease, reaching a minimum in winter (December–March).

### Spectral analysis and LSF analysis

Power spectral densities (PSDs) obtained with the MEM spectral analysis (Additional file 1) for chickenpox data of Denmark (Fig. 1a) and Finland (Fig. 2a) are shown in Figs. 1c and 2c, respectively. In each PSD, prominent spectral lines were observed at *f* = 1.0 [*f* (1/year); frequency] (= *f*_1_) and *f* = 2.0 (= *f*_1_ × 2 = *f*_2_), corresponding to the 1-year cycle and the 6-month cycle, respectively. For investigating the seasonality of each set of chickenpox data (Figs. 1a and 2a) in detail, we calculated the least squares fitting (LSF) curve (Additional file 2) with the 1-year and 6-month cycles that were clearly observed in the PSD (Figs. 1c and 2c). The LSF curves thus obtained are presented in Fig. 1a for Denmark and Fig. 2a for Finland. In both figures, the LSF curves demonstrated a unimodal seasonal cycle. To assign periodic modes constructing the seasonality of the original time series data (Figs. 1a and 2a), we defined a ‘contribution ratio’ (Additional file 3). For Denmark, the contribution ratios of the 1-year cycle (*Q*_1_) and that of the 6-month cycle (*Q*_2_) are 0.71 and 0.003, respectively. For Finland, the *Q*_1_ and *Q*_2_ values were 0.79 and 0.11, respectively. The sum of the *Q*_1_ and *Q*_2_ values for Denmark and Finland was 0.71 (corresponding to 71%) and 0.90 (corresponding to 90%), respectively, indicating that the 1-year and 6-month cycles contributed substantially to the seasonal oscillations.

### Correlation between reported chickenpox cases and temperature

**Fig. 3:**
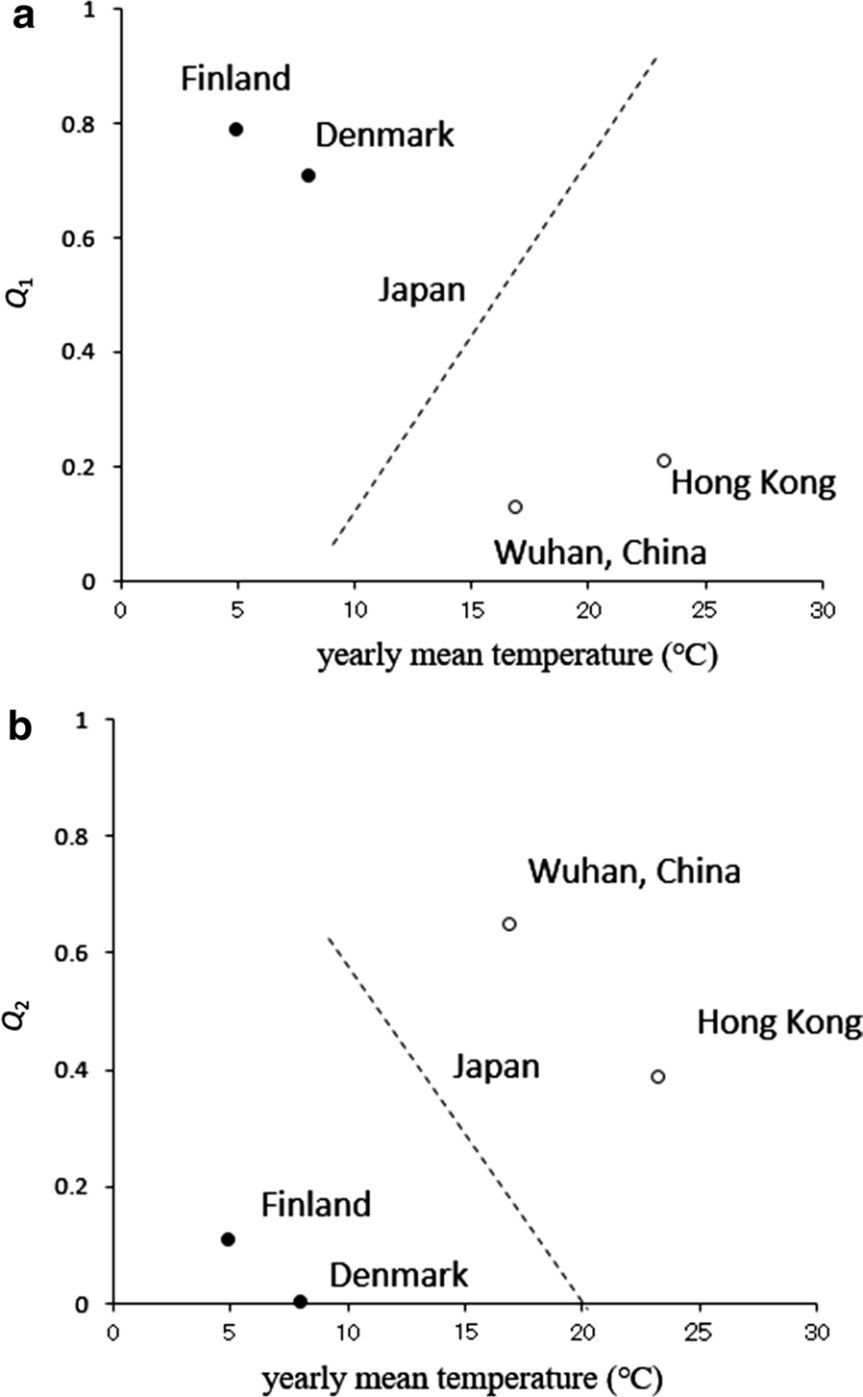
A plot of *Q*_1_ (**a**) and *Q*_2_ (**b**) with filled circles against the mean temperature in Denmark from January 1938 to December 1960 and Finland from January 1952 to December 1970. Dashed lines indicate results from 47 prefectures in Japan (Fig. 6 in Ref. [3]) and open circles indicate the results from Wuhan and Hong Kong (Fig. 4 in Ref. [6]), as shown for comparison with the results from Denmark and Finland reported in this study

Figure 3a, b shows plots of the contribution ratio of the 1-year cycle (*Q*_1_) and the 6-month cycle (*Q*_2_), respectively, to the mean temperature during 1938–1960 for Denmark and during 1952–1970 for Finland. These figures also show the results reported for 47 prefectures of Japan in a previous study [3]. The mean temperature for Denmark was 4.9 °C [12] and that for Finland (Helsinki) was 8 °C [14].

As shown in Fig. 3a, the *Q*_1_ values for Denmark and Finland showed a positive correlation with the mean annual temperatures in Denmark (7.9 °C) and Finland (4.9 °C). This trend was basically the same as in Japan (Fig. 3a), where the value of *Q*_1_ increased as the annual mean temperature increased. The *Q*_1_ values for Denmark and Finland were larger than those for Hokkaido Prefecture in Japan, for which the annual mean temperature (9.2 °C) was the lowest among the other 46 prefectures.

As presented in Fig. 3b, the *Q*_2_ values for Denmark and Finland showed negative correlation with the annual mean temperature for Denmark (7.9 °C) and Finland (4.9 °C). This trend was basically the same as that observed in Japan (Fig. 3b), where the value of *Q*_2_ decreased as the annual mean temperature decreased. The *Q*_2_ values for Denmark and Finland were smaller than those for Okinawa Prefecture in Japan, for which the annual mean temperature (23.0 °C) was the highest among the other 46 prefectures.

### Discussion and conclusions

The temperature-related trends in the *Q*_1_ and *Q*_2_ values for Denmark and Finland (Fig. 3a, b, respectively) support the findings reported by Shoji et al. [15], who indicated that the number of reported cases of chickenpox increased between 5 and 20 °C (i.e., the temperature range at which the chickenpox virus is activated) and decreased below 5 °C and above 20 °C. The occurrence of epidemics is expected to be unimodal in Denmark and Finland, where the temperature falls below 5 °C in winter and rarely exceeds 20 °C in summer; these data confirmed this.

The results of the current study showed that the *Q*_1_ and *Q*_2_ values relating to temperature for Denmark and Finland (Fig. 3a, b, respectively) were basically the same as for Japan [3]. In our preceding study, the bimodal cycles of reported chickenpox incidences that were clearly observed in areas of northern Japan, such as Hokkaido Prefecture (latitude 43°N), disappeared at lower latitudes, and unimodal cycles appeared in Okinawa Prefecture, the southernmost prefecture in Japan (latitude 26°N). This transition of patterns of reported chickenpox incidences in Japan is thought to depend on temperature. Thus, it is reasonable to see that the temporal patterns of chickenpox incidences in Denmark and Finland (Figs. 1a and 2a, respectively) were dominated by temperature as well.

The epidemiology of chickenpox in temperate Denmark may basically be consistent with that in temperate Japan. In a previous study, we found that in Japan, preschool children (age ≤ 4 years) accounted for over 70% of all chickenpox cases in 2000–2011 [3]. Similar to Japan, chickenpox frequently occurs in pre-school children in Denmark (age ≤ 4 years) [12]. However, in Wuhan, China, located in the subtropical zone, chickenpox typically occurs at older ages, with many cases among older schoolchildren and adolescents (age 5–19 years) [7]. We considered that the relatively low number of reported cases of chickenpox among preschool children (age ≤ 4 years) in Wuhan may result from lower chickenpox virus transmission in tropical areas [16]. Accordingly, the effect of meteorological factors on chickenpox incidence may differ from one country to another in different climatic regions.

We confirmed that mean temperature significantly influences the incidence of chickenpox. This factor may be an important predictor of chickenpox incidence in Denmark and Finland. Further time-series analyses of chickenpox and meteorological data of other regions may contribute to determining the disease’s potential relationship with epidemiological factors.

## Limitation

This study was limited in that we did not have access to time series data of chickenpox case notifications in the post-vaccination era in Denmark and Finland. Increased awareness of chickenpox concerning the post-vaccination era in surveillance systems in Denmark and Finland would result in efficient estimation of an effect of vaccination on such infections, so as to prevent chickenpox in these countries.

## Additional files

**Additional file 1.** MEM spectral analysis.

**Additional file 2.** Least squares method (LSM).

**Additional file 3.** Determination of the ‘contribution ratio’.

## Abbreviations

LSF: least squares fitting
MEM: maximum entropy method
PSD: power spectral density
