# Supplementary material for: Role of temperature in reported chickenpox cases in northern European countries: Denmark and Finland
Source: BMC Res Notes. 2018 Jun 13;11:377. doi: 10.1186/s13104-018-3497-0 (PMC5998584; doi:10.1186/s13104-018-3497-0)
Supplement: Supplementary file 1 — Additional file 1. MEM spectral analysis. [file 13104_2018_3497_MOESM1_ESM.pdf]

Additional file 1

MEM spectral analysis

Power spectral density (PSD) based on maximum entropy method (MEM), $P(f)$ (where f represents frequency), for the time series with equal sampling interval Δt, can be expressed by

$$P(f) = \frac{P_m \Delta t}{\left| 1 + \sum_{k=-m}^{m} \gamma_{m.k} \exp\left[-i2\pi fk\Delta t\right] \right|^2}, \qquad \text{(A1)}$$

where the value of P_m is the output power of a prediction-error filter of order m and $\gamma_{m,\,k}$ is the corresponding filter order. The value of the MEM-estimated period of the nth peak component T_n ($=1/f_n$; where f_n is the frequency of the nth peak component) can be determined by the positions of the peaks in the MEM-PSD.
