# Supplementary material for: Role of temperature in reported chickenpox cases in northern European countries: Denmark and Finland
Source: BMC Res Notes. 2018 Jun 13;11:377. doi: 10.1186/s13104-018-3497-0 (PMC5998584; doi:10.1186/s13104-018-3497-0)
Supplement: Supplementary file 2 — Additional file 2. Least squares method (LSM). [file 13104_2018_3497_MOESM2_ESM.pdf]

Additional file 2

Least squares method (LSM)

The validity of the MEM spectral analysis results was confirmed by calculation of the least squares fitting (LSF) curve to the original time series with MEM estimated periods. The formulation of the LSF curve in $X(t)$ is described as follows:

$$X(t) = A_0 + \sum_{n=1}^{N} A_n \cos\{2\pi f_n (t + \theta_n)\}, \qquad \text{(A2)}$$

which is calculated using the LSM for $x(t)$ with unknown parameters f_n, A_0 and A_n (n = 1, 2, 3, …, N), where f_n (=1/T_n; T_n is the period) is the frequency of the n-th component, A_0 is a constant that indicates the average value of the time series data, A_n and θ_n are the amplitude and the phase of the n-th component, respectively, and N is the total number of components.
