# Supplementary material for: Role of temperature in reported chickenpox cases in northern European countries: Denmark and Finland
Source: BMC Res Notes. 2018 Jun 13;11:377. doi: 10.1186/s13104-018-3497-0 (PMC5998584; doi:10.1186/s13104-018-3497-0)
Supplement: Supplementary file 3 — Additional file 3. Determination of the ‘contribution ratio’. [file 13104_2018_3497_MOESM3_ESM.pdf]

Additional file 3

Determination of the 'contribution ratio'

Based on the result of MEM spectral analysis, we assign periodic modes f_n in Eq. (A2) that construct seasonal variations of the original time series $x(t)$. First, the power of each periodic mode is evaluated by the square of amplitude, A_n^2, of the n-th mode constituting the LSF curve. Second, we estimate R corresponding to the power of residual time series, which is obtained by subtracting the LSF curve from the original time series. As a result, the total power of the original time series Q is obtained by

$$Q = A_n^2 + R. \qquad \text{(A3)}$$

When both sides of Eq. (A3) are divided by Q, we obtain the following normalized relationship:

$$A_n^2/Q + R/Q = 1 \qquad \text{(A4)}$$

where A_n^2/Q and R/Q correspond to the contribution of A_n^2 and R to Q, respectively. We define the first term of the left-hand side of Eq. (A4) the 'contribution ratio', which means the

contribution A_n^2 normalized by Q. If A_n^2/Q in the first term becomes large, then the second term, R/Q, becomes small.
